# Single-cell RNA sequencing reveals the epithelial cell, fibroblast, and key gene alterations in chronic rhinosinusitis with nasal polyps

**DOI:** 10.1038/s41598-024-52341-8

**Published:** 2024-01-27

**Authors:** Yakun Wang, Zufei Li, Jun Lu

**Affiliations:** 1 Department of Pathology, Beijing Chaoyang Hospital, Capital Medical University, No. 8 Gongti South Road, Chaoyang District, Beijing, 100020 People’s Republic of China; 2 Department of Otorhinolaryngology, Head and Neck Surgery, Beijing Chaoyang Hospital, Capital Medical University, Beijing, 100020 People’s Republic of China

**Keywords:** Biological techniques, Computational biology and bioinformatics, Molecular biology, Biomarkers, Molecular medicine

## Abstract

Chronic rhinosinusitis with nasal polyps (CRSwNP) is a chronic inflammatory disease of the nasal mucosa, and epithelial–mesenchymal transition (EMT) is thought to be an essential process in the pathogenesis of CRSwNP. However, the mechanisms of epithelial and fibroblastic changes at the single-cell level are unclear. In this study, we investigated the epithelial cell, fibroblast, and key gene alterations in the development of CRSwNP. We revealed major cell types involved in CRSwNP and nasal mucosal inflammation formation, then mapped epithelial and fibroblast subpopulations. We showed that the apical and glandular epithelial cells and the ADGRB3+ and POSTN+ fibroblasts were the key cell subtypes in the progression of CRSwNP. Pseudotime and cell cycle analysis identified dynamic changes between epithelial cells and fibroblasts during its development. WFDC2 and CCL26 were identified as the key marker genes involved in the development of CRSwNP and were validated by IHC staining, which may provide a potential novel target for future CRSwNP therapy. ScRNA-seq data provided insights into the cellular landscape and the relationship between epithelial cells and fibroblasts in the progression of CRSwNP. WFDC2 and CCL26 were identified as the key genes involved in the development of CRSwNP and may be the potential markers for gene therapy.

## Introduction

Chronic rhinosinusitis with nasal polyps (CRSwNP) patients suffer from repeated nasal congestion, purulent secretions, a decreased sense of smell, and life-threatening asthma^1–3^. Due to the high recurrence rate of CRSwNP, patients require long-term medication and repeated surgical treatment, which seriously increases their economic burden and notably affects their quality of life^3^.

The specific mechanism underlying CRSwNP has not yet been fully elucidated. Current studies suggest that epithelial–mesenchymal transition (EMT), the immune system, and pathogens may all play important roles in the pathogenesis of CRSwNP^4^. EMT was first introduced by Hay^5^ and has been proven to play a significant role in wound healing, fibrosis, cancer pathogenesis, and the progenesis of nasal polyps^6,7^. EMT in the nasal mucosa leads to tissue removal, loss of epithelial polarity, downregulation of junctional proteins, and the hyperplasia of mesenchymal cells^7^. EMT is essential for epithelial cells to acquire mesenchymal fibroblast-like characteristics such as reduced intercellular adhesion and increased motility^4,8^. The mesenchymal cells mainly consist of fibroblasts, and their levels increase with CRSwNP, and this is correlated with disease severity^9,10^.

Single-cell RNA sequencing (scRNA-seq), owing to its high accuracy and specificity, has become an ideal tool for single-cell research. It can also be used to discover new cell types, analyze the genome, transcriptome, epigenome, and proteome of a single cell, as well as to determine the cell lineage differentiation track. In recent years, it has been widely used in biomedical research^10^. Existing studies have used scRNA-seq in nasal polyp-related research, but most of these studies have focused on the immunology of CRSwNP^11–13^. The specific mechanism of EMT in CRSwNP has not yet been elucidated using scRNA-seq. In this study, we aimed to investigate the dynamics of epithelial cells and fibroblasts in CRSwNP using scRNA-seq and identify the key genes involved in this process. Our findings reveal the key cell subsets that may be involved in the formation of nasal polyps and nasal mucosa inflammation and identify the key genes involved in the development of CRSwNP, which may serve as molecular targets in future CRSwNP treatments.

## Materials and methods

### Subjects and specimens

This study was approved by the Institutional Review Board of Beijing Chaoyang Hospital Ethics Committee (No. 2020-3-16-18 and date of approval: 2020-3-19). Sample collection and all experiments involving human participants were performed in accordance with institutional ethical regulations and the Declaration of Helsinki ethical principles. All patients provided written informed consent before inclusion in the study. The CRSwNP patients who underwent nasal polyp resection were defined as the CRSwNP group, and the control group included the patients with the deviated septum corrected by surgery. The diagnosis of CRSwNP was made according to the European Position Paper on Rhinosinusitis and Nasal Polyps 2020^14^. Patients with CRSwNP who took oral glucocorticoids, antimicrobials, antileukotrienes, and antihistamines four weeks prior to sample collection were excluded. CRSwNP was divided into eosinophilic chronic rhinosinusitis with nasal polyps (eCRSwNP) and noneosinophilic chronic rhinosinusitis with nasal polyps (neCRSwNP) based on the quantity of eosinophils in nasal polyps. Individual was classified as eCRSwNP were those whose nasal polyp tissue eosinophil number per high-power field (400× magnification) was ten or greater, otherwise as neCRSwNP^11^. Patients with deviated nasal septum who also had asthma, allergic rhinitis, or aspirin sensitivity were not included. According to the inclusion and exclusion criteria, a total of 46 patients were enrolled, including CRSwNP group [n = 23; 3 for scRNA-seq, 20 for immunohistochemistry (IHC)] and control group (n = 23; 3 for scRNA-seq, 20 for IHC). Six out of 46 patients were enrolled, and their samples were prepared for later scRNA-seq studies (CRSwNP group: n = 3; control group: n = 3). Specifically, all 3 patients in the CRSwNP group were eCRSwNP. The samples were collected: mucosal tissues of nasal polyps (NP group, n = 3) and nasal mucosa tissues adjacent to nasal polyps (UT group, n = 3) from the eCRSwNP; nasal mucosa of uncinate tissues (CM group, n = 3) from the control group. The abnormal uncinate anatomy of deviated nasal septum needed to be corrected to improve the ventilation^15^. These samples were prepared for later scRNA-seq studies. Details of clinical characteristics were summarized in Supplementary Table 1.

### Single-cell suspension preparation and quality control

Based on the source and characteristics of the tissue or cell samples, the concentration and quality of the single-cell suspensions were strictly controlled, and the qualified samples were used for 10× single-cell transcriptome sequencing. Cell suspension quality requirements were as follows: total number of cells > 500,000, cell activity > 85%, cell concentration 700–1200 cell/μL, cell volume > 100 μL, clumping rate < 15%, cell diameter < 40 μm, absence of reverse transcription inhibitors, and non-cellular nucleic acid molecules.

### Sequencing library preparation and single-cell RNA-seq data preprocessing

We used Chromium Next GEM Single Cell 3ʹ Reagent Kits v3.1 on the Chromium Controller (10× Genomics) to prepare single-cell RNA-seq libraries, The Cell Ranger software called STAR^16^ was used to analyze the sequencing data. The detail of Sequencing library preparation and Single-cell RNA-seq data preprocessing were shown in supplementary materials.

### Clustering

The Seurat (4.0.1) software^17^ was used to cluster the data, the principal component analysis (PCA) method was used to reduce the dimensionality of the data. T-distributed Stochastic Neighbor Embedding (TSNE) with Uniform Manifold Approximation and Projection (UMAP) were used for visualization and presentation. SingleR (v1.4.1) was used for automatic annotation of the cellular taxa and manual annotation corrections. We further corrected the cell types using the Cell Marker database (http://bio-bigdata.hrbmu.edu.cn/CellMarker/).

### Marker gene analysis

A fold change ≥ 2 and threshold value < 0.1 (parameter is: FDR) were used as the screening criteria, and the most significantly differentially expressed genes in each cluster were selected as the marker genes. The differential genes were compared using the SwissProt^18^, GO^19^, COG, KOG, Pfam, KEGG^20^, and Reactome^21^ databases for sequencing to obtain gene annotation information. The CM group vs. UT group and UT group vs. NP group were each compared to detect the differences in the differential gene levels between groups.

### Pseudotime analysis

Monocle 2 (v2.18.0), a reversed graph embedding (RGE) learning approach, was used to predict and reconstruct cell developmental differentiation trajectories based on cellular gene expression information. We used Monocle 2 (v2.18.0) to analyze and reconstruct complex differentiation trajectories of epithelial cells and fibroblasts by placing cells onto pseudotime trajectories (https://github.com/cole-trapnell-lab/monocle-release).

### Cell cycle analysis

The cell cycle encompasses the entire process of cell division, from the completion of one division to the end of the subsequent division. G1 phase is the preparation phase for DNA synthesis, S phase is the DNA synthesis phase, G2 phase is the preparation phase for division, and M phase is the division phase. Single-cell transcriptome data were analyzed using the Seurat “AddModuleScore” function for cell cycle analysis. Based on Seurat's built-in cycle signature proteins, the transcriptional expression of the cycle signature proteins in each cell was calculated. The possible cycle status of each cell type was scored to determine whether the cell was in a proliferative state.

### Immunohistochemistry (IHC) staining analysis

Then scRNA-seq results was validated by IHC. Forty patients were enrolled, including the CRSwNP group (n = 20) and control group (n = 20) for IHC validation. There were 11 cases with eCRSwNP and 9 with neCRSwNP in the CRSwNP group. Specifically, the samples of mucosal tissues of nasal polyps (NP group, n = 20) and nasal mucosa tissues adjacent to nasal polyps (UT group, n = 20) from the CRSwNP group, and nasal mucosa (CM group, n = 20) of uncinate tissues from the control group were collected and processed for IHC experiments to further verify the above result. Details of clinical characteristics were summarized in Supplementary Table 2. The tissues were dehydrated and embedded, and tissue sections were cut using a microtome, mounted on slides, and dried at 65 °C for 1 h. The tissue sections were then dewaxed using xylene and ethanol and then immersed in citrate solution and heated in a microwave oven for 15 min. Blocking was performed using bovine serum albumin to avoid non-specific antibody binding. Tissue sections were incubated with primary antibodies (anti-WFDC2, dilution 1:500; anti-CCL26, dilution 1:350; Abcam, Cambridge, USA) in a humidified chamber overnight at 4 °C. The secondary antibody was added and incubated for 40 min at room temperature. Positive rate analysis of the IHC staining was performed using ImageJ software (NIH, Bethesda, MD, USA).

### Statistical analysis

Spearman correlation analysis was performed to analyse the association between the expression of WFDC2 and CCL26.To compare the expression levels of key genes among the CM, UT, and NP groups, Student's t test was used to determine the significance of differences between samples. All *P* values were two-tailed, with a *P* value less than 0.05 considered statistically significant. Statistical analyses were conducted using SPSS 21 statistical software (SPSS, Chicago, IL, USA) or Prism 9.0 software (GraphPad software Inc. CA, USA).

## Results

### Cell profiling of the CM, UT, and NP tissues using single-cell RNA sequencing

**Figure 1:**
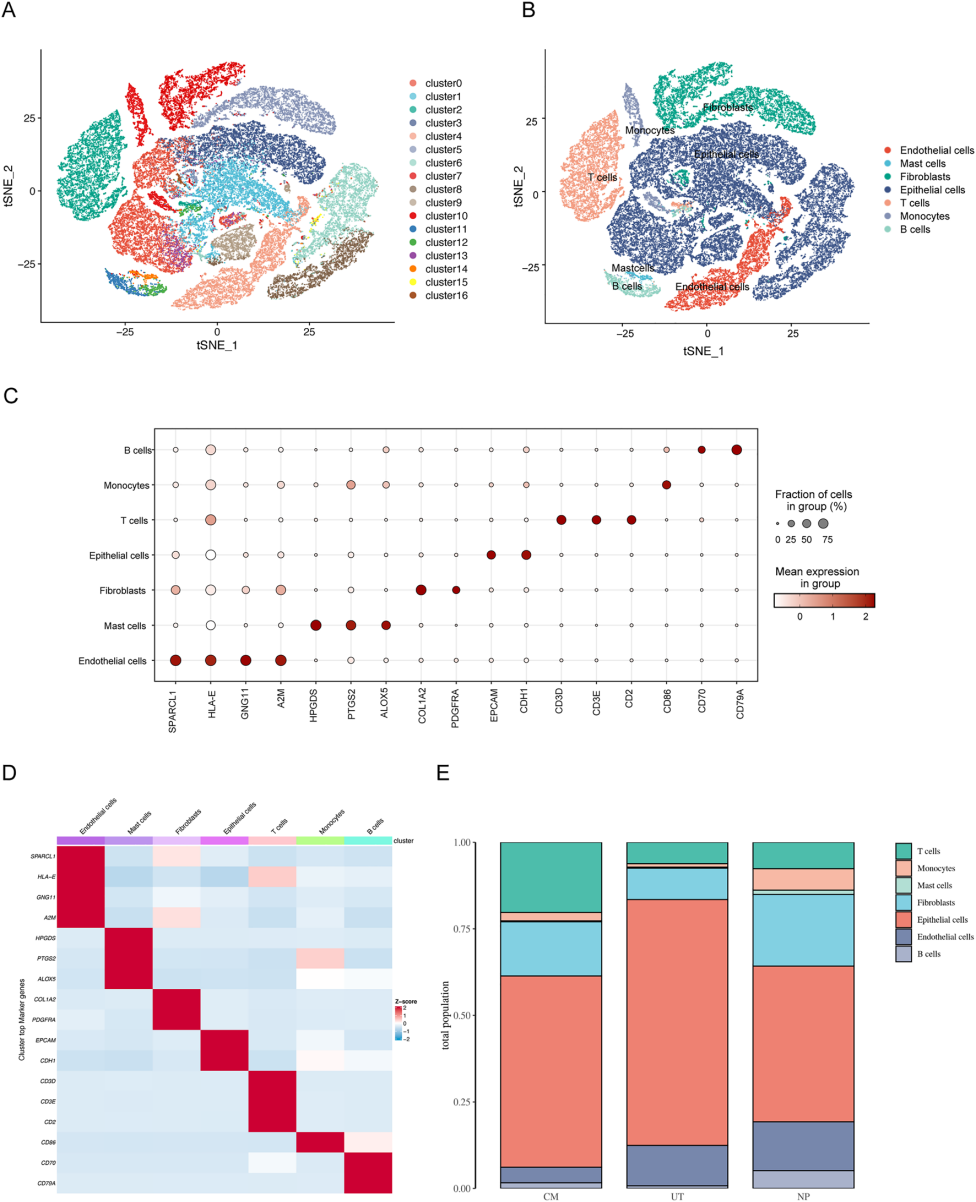
Cell profiling of the CM, UT, and NP tissues. (**A**) Seventeen cell clusters were grouped using TSNE. (**B**) Seven types of cell groups were distinguished, as follows: endothelial cells, mast cells, fibroblasts cells, epithelial cells, T cells, monocytes, and B cells. (**C**,**D**) Bubble chart and heat diagram for each mark gene and cluster. (**E**) Distribution of each cell group in the different tissue types.

To explore how normal nasal mucosa could cause chronic inflammation and form nasal polyps, nine tissues were obtained and analyzed; these included three nasal polyp samples (NP group), three uncinate tissue samples that were adjacent to the nasal polyps (UT group), and three control nasal mucosa samples from patients diagnosed with a deviated nasal septum (CM group). 47,908 highly qualified cells were identified, 17 cell clusters were identified (Fig. 1A) and 7 cell group types were distinguished (Fig. 1B), which were as follows: endothelial cells (*SPARCL1, HLA-E, GNG11, A2M*), mast cells (*HPGDS, PTGS2, ALOX5*), fibroblasts cells (*COL1A2, PDGFRA*), epithelial cells (*EPCAM, CDH1*), T cells (*CD3D, CD3E, CD3D2*), monocytes (*CD86*), and B cells (*CD70, CD79A*) (Table 1). A bubble chart and heat diagram for each marker gene and the clustered cells are shown in Fig. 1C,D, respectively. The distribution of each cell group in the different tissue types is shown in Fig. 1E. The NP group had a lower proportion of epithelial cells and more fibroblasts than the UT or CM group.

**Table 1:** Summary of the cell clusters.

Cluster	Key genes	Number of cell captured									Putative identity
		CM1	CM2	CM3	UT1	UT2	UT3	NP1	NP2	NP3	
C0	EPCAM and CDH1	600	169	1468	1032	1286	818	766	304	39	Epithelial cells
C1		158	83	408	1736	174	322	2608	169	477	
C3		482	291	1326	929	988	955	463	249	18	
C6		1713	332	920	168	85	12	208	37	154	
C8		433	165	570	306	87	37	657	132	316	
C9		110	77	310	93	286	378	207	104	24	
C13		38	8	67	4	233	59	25	56	32	
C15		112	17	13	14	4	30	0	1	0	
C16		27	26	4	17	31	10	8	3	0	
C2	CD3D, CD3E and CD2	2312	945	383	465	204	205	755	266	183	T cells
C5	CD70 and CD79A	510	450	766	116	166	32	1144	539	444	Fibroblasts
C7		319	205	565	229	338	402	576	234	332	
C4	SPARCL1, HLA-E, GNG11 and A2M	284	156	369	760	421	465	1297	404	524	Endothelial cells
C10	CD86	205	148	84	70	65	10	680	98	202	Monocytes
C11	CD70 and CD79A	43	40	128	66	8	1	286	17	49	B cells
C12		29	48	7	22	7	6	271	47	145	
C14	HPGDS, PTGS2 and ALOX5	12	14	23	136	16	44	26	6	6	Mast cells

### Subgroup cluster results for the epithelial cells

**Figure 2:**
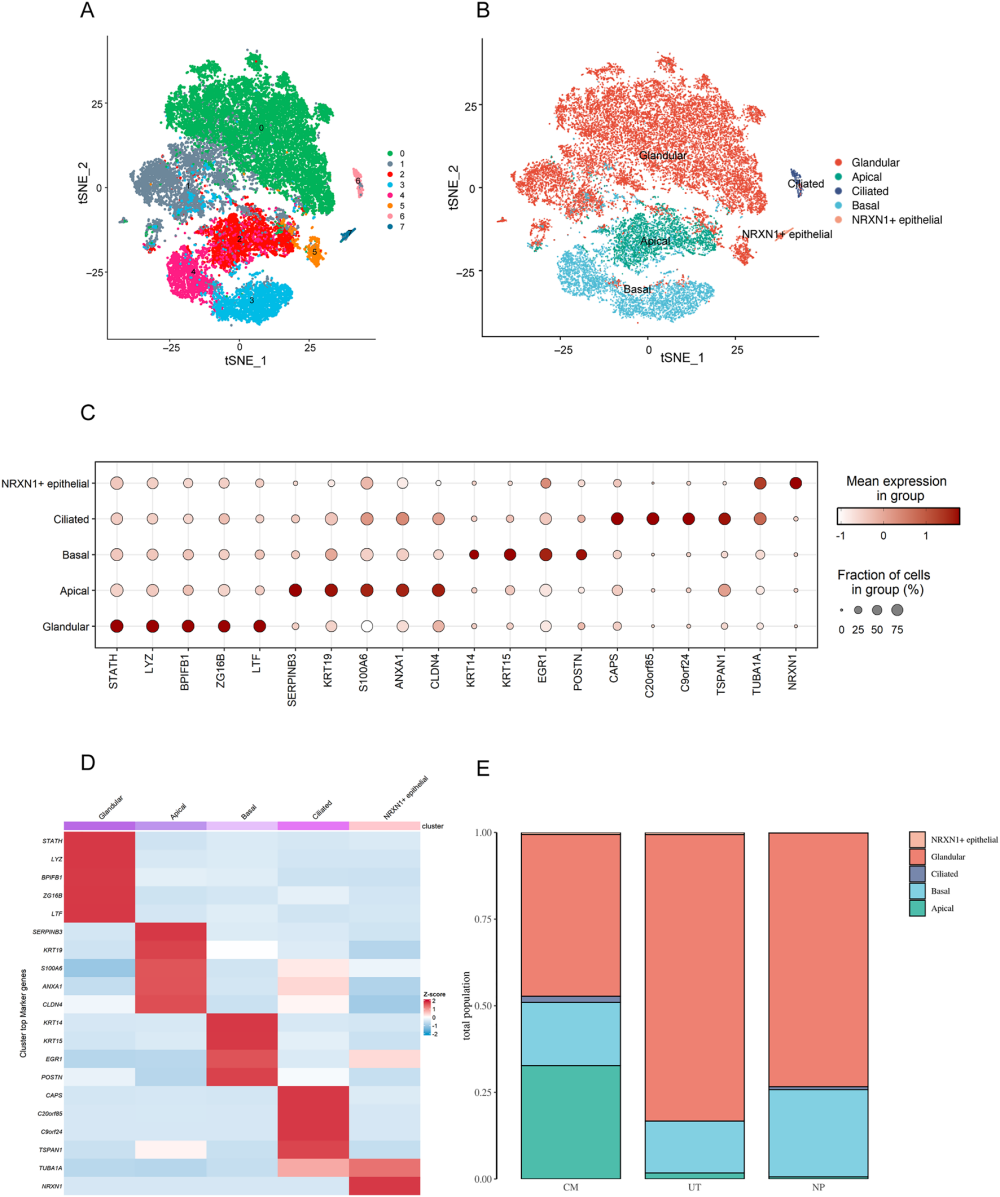
Epithelial cell subgroups cluster results. (**A**) Epithelial cells are clustered into eight subgroups. (**B**) The five epithelial cell categories identified were as follows: glandular cells, apical cells, ciliated cells, basal cells, and NRXN1+ cells. (**C**,**D**) Bubble chart and heat map for each mark gene and cluster. (**E**) Distribution of each cell group in the different tissue types.

To explore the composition of the epithelial cells, they were clustered using TSNE. The epithelial cells were clustered into 8 subgroups (Fig. 2A) and 5 categories were identified (Fig. 2B), including glandular cells (*STATH, LYZ, BPIFB1, ZG16B, LTF*), apical cells (*SERPINB3, KRT19, S100A6, ANXA1, CLDN4*), ciliated cells (*KRT14, KRT15, EGR1, POSTN*), basal cells (*CAPS, C20orf85, C9orf24, TSPAN1, TUBA1A*), and NRXN1+ cells (*NRXN1*). Bubble charts and heat maps for each marker gene and cluster are shown in Fig. 2C,D, respectively. The distribution of each cell group in the different tissue types is shown in Fig. 2E. The proportion of apical cells was highest in the CM group, followed by the UT group, and lowest in the NP group. The proportion of apical cells in the NP and UT groups was much lower than that in the CM group, indicating that the apical cells were destroyed or differentiated into other cells during inflammation and the formation of nasal polyps. The glandular cells were the most abundant in the UT group, followed by the NP and CM groups, indicating that the epithelium of the non-polyp mucous membrane tissues of the patients with CRSwNP secreted significantly more mucus than that of the mucous membrane tissues of the control group patients. No considerable differences among the groups were found for the other epithelial cell subtypes.

### Fibroblast subgroup cluster results

**Figure 3:**
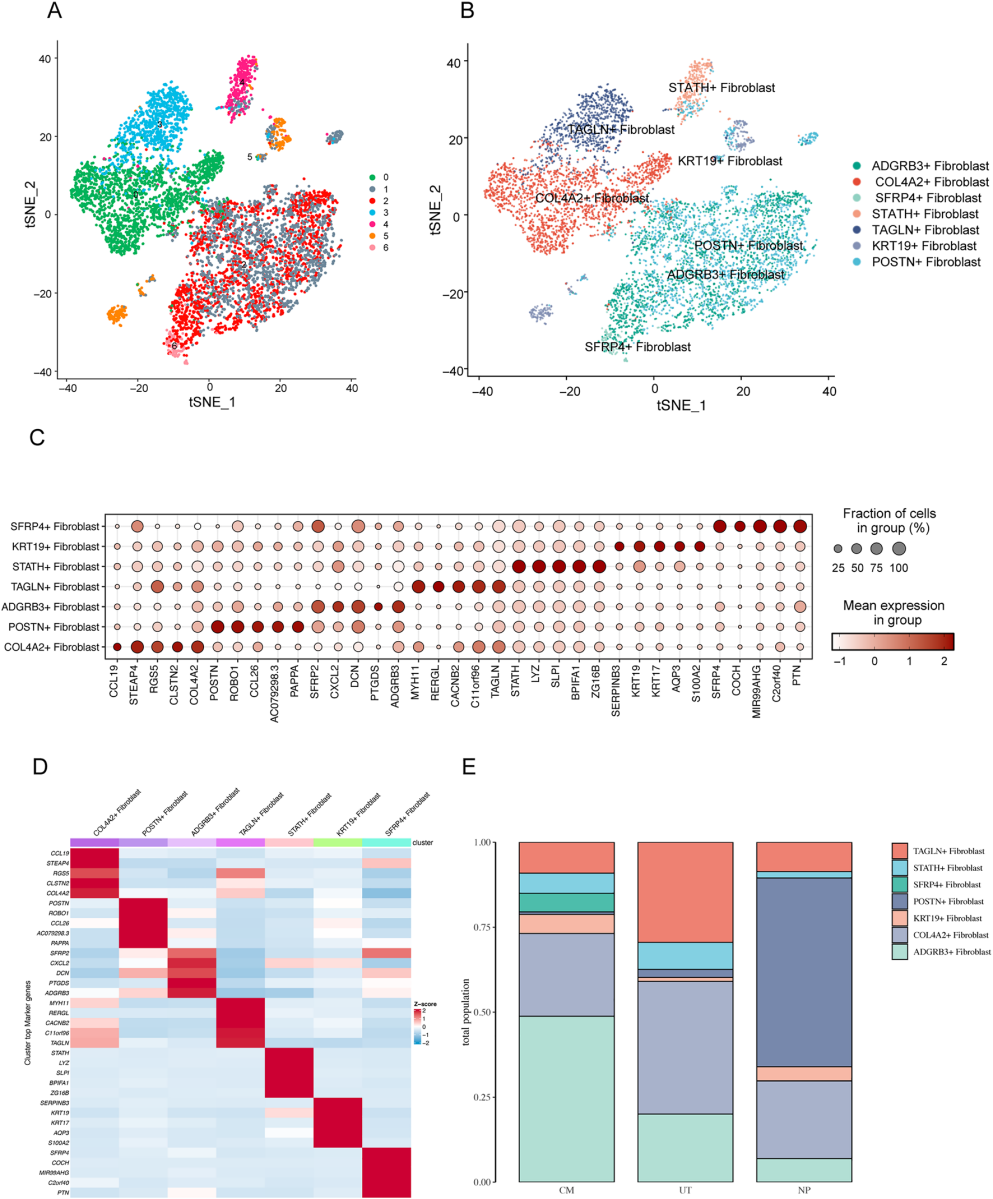
Fibroblast cell subgroups cluster results. (**A**) Fibroblasts are clustered into seven categories. (**B**) The seven cell subgroups identified were as follows: ADGRB3+ fibroblasts, COL4A2+ fibroblasts, SFRP4+ fibroblasts, STATH+ fibroblasts, TAGLN+ fibroblasts, KRT19+ fibroblasts, and POSTN+ fibroblasts. (**C**,**D**) Bubble chart and heat map for each mark gene and clustered cells. (**E**) Distribution of each cell group in the different tissue types.

Cell clustering was performed to find highly variable genes using the function FindVariableFeatures, which was calculated using vst. Highly variable genes were the parameters: 2000, and then standardized analysis was done. Fibroblasts were used to make TSNE figures according to the cluster using 0.16 resolution. We divided the fibroblasts into seven clusters. The fibroblasts were clustered into 7 categories (Fig. 3A) and were annotated into the 7 cell subgroups according to their most differential genes (Fig. 3B) as the following: ADGRB3+ fibroblasts (*SFRP2, CXCL2, DCN, PTGDS, ADGRB3*), COL4A2+ fibroblasts (*CCL19, STEAP4, RGS5, CLSTN2, COL4A2*), SFRP4+ fibroblasts (*SFRP4, COCH, MIR99AHG, C2orf40, PTN*), STATH+ fibroblasts (*STATH, LYZ, SLPI, BPIFA1, ZG16B*), TAGLN+ fibroblasts (*TAGLN, C11orf96, CACNB2, RERGL, MYH11*), KRT19+ fibroblasts (*SERPINB3, KRT19, KRT17, AQP3, S100A2*), and POSTN+ fibroblasts (*POSTN, ROBO1, CCL26, AC079298.3, PAPPA*). Bubble diagrams and heat maps for each marker gene and cluster were shown in Fig. 3C,D, respectively. The distributions of the various cell groups in the different tissues were shown in Fig. 3E.

ADGRB3+ fibroblast levels were highest in the tissues of the control group, followed by the UT and NP groups, suggesting that this cell subgroup type may be a protective factor against the occurrence and development of nasal polyps, similar to tumor suppressor genes. POSTN+ fibroblasts showed a gradually increasing trend among the CM, UT, and NP groups, indicating that the two cell types (POSTN+ fibroblasts and ADGRB3+ fibroblasts) may play a mutual role in the occurrence and development of nasal polyps.

### Pseudotime analysis of the epithelial cells and fibroblasts

**Figure 4:**
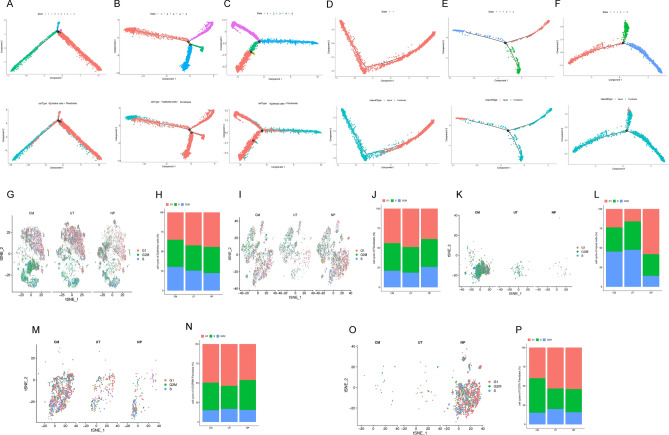
Pseudotime analysis of the epithelial cells and fibroblasts. (**A**–**C**) Pseudotime analysis of the epithelial cells and fibroblasts in the CM, UT, and NP groups, respectively. (**D**–**F**) Pseudotime analysis of the apical cells and fibroblasts in the CM, UT, and NP groups, respectively. Each point in the figure represents a cell, different branches represent different evolution or differentiation tracks, and the horizontal and vertical coordinates represent the components of DDRTree after dimensionality reduction. (**G**,**H**) Cell cycle analysis of the epithelial cells. Cell cycle analysis of the (**I**,**J**) fibroblasts, (**K**,**L**) apical cells, (**M**,**N**) ADGRB3+ fibroblasts cells, and (**O**,**P**) POSTN+ fibroblasts cells.

Monocle was used to conduct the pseudotime analysis and predict and reconstruct the cell development and differentiation trajectory according to information on cell gene expression. The transcriptional state in this trajectory showed normal differentiation pathways and changes associated with the progression of nasal polyp formation. As seen in Fig. 4A, epithelial cells and fibroblasts are located on different branches of the track, marking their various states of differentiation. Compared to the CM and UT group (Fig. 4A,B), the NP group (Fig. 4C) showed a trend that part of the epithelial cells was differentiated into fibroblasts. Pseudotime analysis between the apical cells and fibroblasts was also conducted and indicated that the apical cells may be partially transformed into fibroblasts during the pathogenesis and development of chronic sinusitis or even the formation of nasal polyps (Fig. 4D–F).

### Cell cycle analysis of the epithelial and fibroblasts cells

The cell cycle status of the epithelial cells and fibroblasts in the UT group did not differ significantly when compared to that in the CM group. However, the proportion of epithelial cells in the G2M state decreased, whereas the proportion of cells in the G1 stage increased in the NP group (Fig. 4G,H); additionally, the proportion of fibroblasts in the G2M state increased. The proportion of cells in the G1 phase decreased (Fig. 4I,J), indicating a decrease in the proliferation capacity of epithelial tissues and an increase in the proliferation capacity of the fibroblasts in the NP group, which further corroborated and elucidated the mechanism by which epithelial tissues decreased, and fibroblast tissues increased in the NP group. Because the epithelial cell subpopulation changed in the three groups, mainly for apical cells, an apical cell cycle analysis was performed. The apical cell cycle analysis showed no significant differences in the cycle status of the apical cells between the UT and CM groups. However, in the NP group, the proportion of apical cells with G2M status decreased, whereas the proportion of G1 phase cells increased (Fig. 4K,L). This indicated that the proliferation capacity of the apical cells in the NP group was reduced. In the CM, UT, and NP groups, no significant changes in the ADGRB3+ fibroblast cell cycles were identified, suggesting that their decline during the formation of CRSwNP might not be due to a decrease in their proliferative capacity (Fig. 4M,N). No significant changes were found in the POSTN+ fibroblast cell cycles, suggesting that their proliferation during the formation of CRSwNP was not necessarily due to an increase in cell proliferation capacity but possibly due to epithelial cell transformation (Fig. 4O,P).

### Differential gene analysis of the epithelial cells in each group

**Figure 5:**
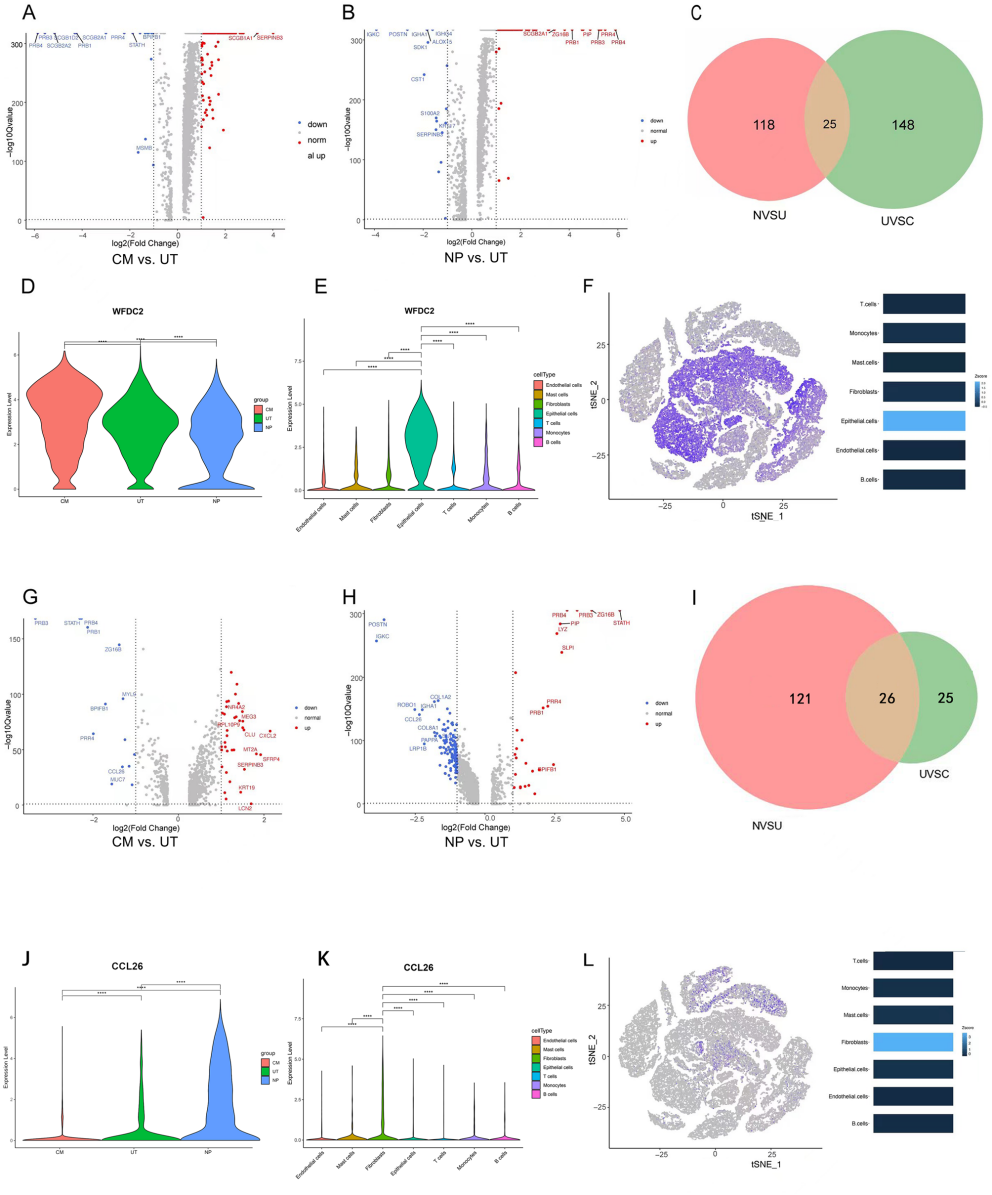
Differential gene analysis of the epithelial cells and fibroblasts in each group. Volcano plots showing the differential genes (**A**) between the CM and UT groups (173 genes), and (**B**) between the NP and UT groups (143 genes). (**C**) There were 25 common differential genes identified in the Venn diagram, NVSU: NP VS UT; UVSC: UT VS CM. (**D**) Expression of the WFDC2 gene in the epithelial cells from the CM, UT, and NP tissues. (**E**) WFDC2 expression in the epithelial cells was much greater than in the other cell types. (**F**) Distribution of the WFDC2 in the TSNE, WFDC2 was mainly expressed in the epithelial cells. Volcano plots showed the differential genes between the (**G**) CM and UT groups and (**H**) the NP and UT groups. (**I**) There were 26 common differential genes identified in the Venn diagram, NVSU: NP VS UT; UVSC: UT VS CM. (**J**) CCL26 expression in the fibroblasts from the CM, UT, and NP tissues. (**K**) CCL26 expression in the fibroblasts was much greater than in the other cell types. (**L**) Distribution of the CCL26 in the TSNE, CCL26 was mainly expressed in the fibroblast cells. *< 0.05, **< 0.01, ***< 0.001, ****< 0.0001.

We would like to investigate the gene expression with continuous trend in epithelial cells from normal nasal mucosa to nasal mucosa tissues adjacent to nasal polyps, further to mucosal tissues of nasal polyps. Therefore, we made comparison of CM vs. UT and NP vs. UT groups to find differential genes. In epithelial cells, we identified 73 differential genes by CM vs. UT groups and 143 by NP vs. UT groups (Fig. 5A,B, respectively). Firstly, we identified 25 co-expressed genes by Venn diagram analysis (Fig. 5C) These 25 genes differed in CM vs. UT and NP vs. UT groups, showing significant changes. Among these genes, we further searched for the genes related to inflammation progression. We found that only the expression of the WFDC2 among the CM, UT, and NP groups decreased in a stepwise manner (CM > UT > NP, Fig. 5D) and was mainly expressed in epithelial cells (Fig. 5E). With the aggravation of inflammation, the expression of WFDC2 decreased. Only this gene was associated with the state of inflammatory progression. The expression of WFDC2 in the epithelial cells was much higher than in the other cell types. WFDC2 in the TSNE was found to be mainly expressed in the epithelial cells (Fig. 5F).

### Differential gene analysis of the fibroblast cells in each group

Similarly, the analytic process was conducted to find the gene expression with continuous trend in fibroblast cells. We identified 51 and 148 differential genes by CM vs. UT and NP vs. UT groups, respectively (Fig. 5G,H). A total of 26 genes were co-expressed by Venn diagram analysis (Fig. 5I) Only the expression of CCL26 among the three groups increased in a stepwise manner (CM < UT < NP, Fig. 5J). Its expression increased with the severity of inflammation. CCL26 was mainly expressed in fibroblasts, as shown in Fig. 5K, and the expression level was much higher when compared with that of other cell types. The distribution of CCL26 in the TSNE (Fig. 5L) showed that it was predominately expressed in fibroblasts.

### Correlation analysis of the WFDC2 and CCL26 genes

**Figure 6:**
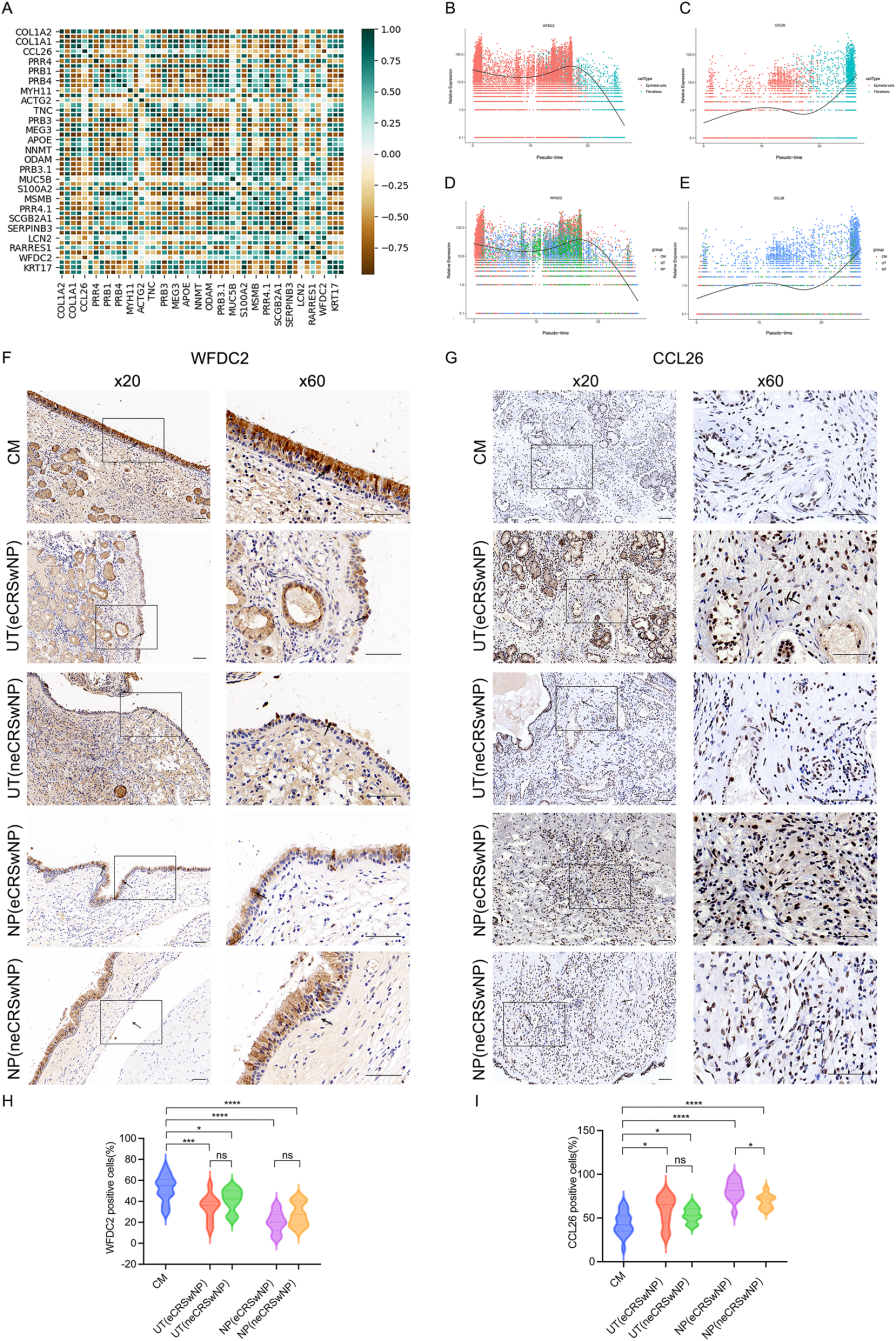
WFDC2 and CCL26 gene correlation analysis. (**A**) Spearman correlation analysis shows that the WFDC2 and CCL26 expression levels were significantly negatively correlated. (**B**) WFDC2 was predominately detected in the epithelial cells, (**C**) CCL26 was predominately detected in the fibroblasts, (**D**) WFDC2 was predominately identified in the CM group, and (**E**) CCL26 was predominately identified in the NP group. (**F**) WFDC2 levels in the CM, UT (eCRSwNP), UT (neCRSwNP), NP (eCRSwNP) and NP (neCRSwNP) groups were analyzed using IHC staining. The boxes show high-power images (×60) obtained from low-power images (×20). The black arrows indicate WFDC2. The scale bars represent 100 μm. (**H**) Unpaired t-tests were used to determine the significant differences for WFDC2 expression among the CM (n = 20), UT (eCRSwNP, n = 11), UT (neCRSwNP, n = 9), NP (eCRSwNP, n = 11) and NP (neCRSwNP, n = 9) groups. (**G**) CCL26 levels in the CM, UT (eCRSwNP), UT (neCRSwNP), NP (eCRSwNP) and NP (neCRSwNP) groups were analyzed using IHC staining. The boxes show high-power images (×60) obtained from low-power images (×20). The black arrows represent CCL26. The scale bars represent 100 μm. (**I**) Unpaired t-tests were used to analyze the significant differences for CCL26 expression among the CM (N = 20), UT (eCRSwNP, n = 11), UT (neCRSwNP, n = 9), NP (eCRSwNP, n = 11) and NP (neCRSwNP, n = 9) groups. *< 0.05, **< 0.01, ***< 0.001, ****< 0.0001.

Spearman correlation analysis elucidated the relationship between the co-expressed genes, as mentioned above. As can be seen, the expression levels of WFDC2 and CCL26 were significantly negatively correlated (Fig. 6A), with a correlation coefficient of − 0.867, P < 0.01. Gene-specific time series analysis revealed that WFDC2 was less involved in fibroblast function and was predominately identified in epithelial cells, whereas CCL26 was predominately identified in fibroblasts (Fig. 6B,C). During the transformation of epithelial cells into fibroblasts, the gene expression level of WFDC2 gradually decreased whereas that of CCL26 showed an obvious upward trend. Pseudotime analysis of the CM and NP groups showed that the expression of WFDC2 gradually decreased, with increased expression in the CM group, and that the lowest expression level was in the NP group. The pseudotime analysis from the CM to NP groups showed that the expression of *CCL26* gradually increased, with the lowest expression in the CM group and the highest expression in the NP group (Fig. 6D,E). These two genes might play key roles in the transformation of epithelial cells and fibroblasts during the formation of CRSwNP.

### Immunohistochemical analyses

IHC analysis of WFDC2 and CCL26 protein expression in the CM, UT, and NP groups was performed. In the CM group, apical cells showed diffuse positive expression in the normal mucosal epithelium. In the UT (eCRSwNP and neCRSwNP) groups, WFDC2 was presented scattered expression in the mucosa epithelium adjacent to nasal polyps. In the NP (eCRSwNP and neCRSwNP) groups, WFDC2 was expressed in individual cells in the mucosal epithelium of nasal polyps (Fig. 6F). The positive rate of WFDC2 in UT (P < 0.001) and NP (P < 0.0001) groups of eCRSwNP was significantly lower than that in CM group. The positive rate of WFDC2 in UT (P < 0.05) and NP (P < 0.0001) groups of neCRSwNP was significantly lower than that in CM group (Fig. 6H). The expression of CCL26 was scattered in the interstitium of CM group. CCL26 was partially expressed in the interstitium of UT group. CCL26 was diffusely positive in the interstitium of NP group (Fig. 6G). The positive rate of CCL26 in UT (P < 0.05) and NP (P < 0.0001) groups of eCRSwNP was significantly higher than that in CM group. The positive rate of CCL26 in UT (P < 0.05) and NP (P < 0.0001) groups of neCRSwNP was higher than that in CM group. The positive rate of CCL26 in eCRSwNP was higher than neCRSwNP in NP group (P < 0.05) (Fig. 6I).

## Discussion

In this study, scRNA-seq technology was used to explore the relationship between epithelial cells and fibroblasts in CRSwNP. The epithelial cell and fibroblast subtypes were identified, and the dynamics between the two cell types were determined. The pseudotime analysis showed that the epithelial cells tended to differentiate into fibroblasts over time. The results also indicated that the apical cells, ADGRB3+ fibroblasts, and POSTN+ fibroblasts may play a significant role in the development of CRSwNP. The cell proliferation was further investigated using cell cycle analysis, and the proliferation ability of the epithelial cells was found to decrease in CRSwNP. WFDC2 and CCL26 were also identified as key genes involved in the pathogenesis of CRSwNP, and a decrease in WFDC2 expression was correlated with an increase in CCL26.

Compared to that in the UT and CM tissues, the proportion of epithelial cells and fibroblasts was significantly decreased and increased, respectively, in the NP tissues. This may be because during NP formation, the epithelial cells are transferred to fibroblast cells, which is also known as EMT^22,23^. Pseudotime analysis showed a trend of transiting into fibroblasts from epithelial cells, especially in the NP group. The proportion of epithelial cells was higher whereas that of fibroblasts was lower in the UT group than in the CM group. This indicated that when the epithelial barrier was damaged and the nasal mucosa was inflamed without polyps, the epithelial cells might be the first to proliferate. With increased damage, epithelial cells became decompensated or transformed into fibroblasts and the number of these cells decreased significantly. E-cadherin was essential for maintaining the epithelial phenotype of cells^24^. Vimentin was a widely used phenotypic marker for the identification of fibroblasts^25^. Our previous study found that the epithelial marker (E-cadherin) was decreased, and the fibroblast marker (vimentin) was increased in CRSwNP^15^. Combined with these results of the study, it was speculated that there might exist a tendency for epithelial cells to differentiate into fibroblasts in CRSwNP. Previous studies have shown that epithelial–mesenchymal transition (EMT) plays a vital role in the occurrence of CRSwNP^26^. Epithelial cells and fibroblasts were important cell types in the occurrence of EMT. This study aimed to investigate the role of EMT in CRSwNP. EMT was an essential process in cell development in which epithelial cells acquired mesenchymal fibroblast-like characteristics and promoted the progression of inflammation^8^. Therefore, in CRSwNP this study mainly focused on the epithelial cells and fibroblasts and analyzed the dynamics between epithelial cells and fibroblasts in three different states: nasal polyps, uncinate tissue adjacent to nasal polyps, and control nasal mucosa from patients with deviated nasal septa.

The epithelial cells were further divided into five sub-groups, and the proportion of glandular cells was higher in both the UT and NP tissues than in the CM tissues. This explains why patients with sinusitis have many nasal secretions, as there are a large number of secretory cells in the nasal polyps and mucosa tissues adjacent to polyps. The apical cells, whether in the NP or UT tissues, were also found to show a precipitous downward trend when compared with those in the normal nasal mucosa tissues, and the apical cell content in the NP tissue was less than that in the UT tissue. Apical cells are on the apical surface of epithelium cells, and they play an important role in forming an epithelial barrier. The decrease in these cells further confirmed that the epithelial barrier function was damaged in nasal polyps and tissues adjacent to polyps, and this increased the permeability of the epithelial cells and the penetration of microorganisms and antigens, causing inflammation and CRS^27^. In addition, owing to the decrease in essential cells, the acute to basic transient pressure increases. These mechanical responses of cells to pressure may activate the mechanical transduction pathway that drives cytoskeleton remodelling, further increasing the possibility of tissue remodelling^28,29^.

Fibroblasts are critical components of the nasal cavity and sinus mucosa. Chronic nasal mucosal inflammation guides epithelial remodelling through the secretion of inflammatory mediators. Fibroblasts in nasal polyps can produce a variety of inflammatory mediators that promote eosinophil infiltration and chemokine secretion to recruit macrophages that will stimulate the continuous production of nasal secretions. Fibroblasts promote the formation of nasal polyps^9^. In-depth analyses of the fibroblast subsets were not conducted in previous studies as fibroblasts have generally been considered as a single cell group. In this study, the fibroblast cells were divided into 7 sub-groups, and the results showed that ADGRB3+ fibroblast cells were the highest in the CM group, followed by the UT group and NP group, with significant differences. This indicated that these cells may have a protective function during the pathogenesis of NP. ADGRB3 is also known as adhesion G protein-coupled receptor B3 (BAI3)^29^, which is a member of the cell adhesion group of the G protein-coupled receptor (GPCR)^30^. It was speculated that ADGRB3 may be related to cell adhesion and that its reduction leads to a decrease in cell adhesion^31^, which subsequently triggers the damage of epithelial barrier function. The lineage transition from epithelium to mesenchyme is a process known as EMT, by which polarized epithelial cells lose their adhesion property and obtain mesenchymal cell phenotypes^32^. Xia et al. found that EMT was widespread in the nasal mucosa of CRSwNP and involved in the pathogenesis of CRSwNP^26^. Therefore, ADGRB3 might correlate with EMT to promote the occurrence of CRSwNP.

In contrast to the proportion of ADGRB3+ cells, that of POSTN+ cells in the CM, UT, and NP tissues increased successively in the fibroblast cells. POSTN is an extracellular matrix protein (ECM) that is essential for EMT in a variety of tumour cells^33^. POSTN was overexpressed in adamantinomatous craniopharyngioma (ACP)-associated fibroblasts^33^. The overexpression of POSTN has also been verified to promote EMT in renal cell carcinoma cells by activating the IKL/AKT/mTOR pathway^34^. Bronchial biopsies confirmed higher levels of POSTN protein expression in the epithelium of patients with asthma than those in that of healthy controls^35^. POSTN induced EMT to promote the migration and invasion of ovarian cancer cells^36^. Previous studies have shown that the expression of POSTN in NP groups is much higher than that in normal nasal mucosa tissues, indicating that this gene is a pathogenic factor of CRSwNP^37^. EMT was widely present in the nasal mucosa of CRSwNP and contributes to the pathogenesis of the disease^26^. It was speculated that the increase in POSTN+ cells is one of the pathogenic factors involved in CRSwNP formation. The results showed that POSTN can be used as a genetic marker for one of the fibroblast subsets in CRSwNP, indicating that this gene may play a main role in this fibroblast subgroup.

The low inflammation uncinate process was the transition status in the process of nasal polyps formation^38^. Additionally, nasal polyps had a greater inflammatory infiltration than the nasal mucosa tissues adjacent to nasal polyps^39^. The low inflammation status of uncinate process described in this study was to observe the trend of dynamic inflammatory changes in the progression of CRSwNP and explain the occurrence of CRSwNP. In the study, the clear differences were found in comparisons of NP and normal epithelium at the level of the epithelium and fibroblast functions. These were complemented by a description of altered cell cycle dynamics, with G2 reduction and G1 increase. The results of the cell cycle analysis showed that, when compared with those of the CM and UT tissues, the epithelial cells of NP tissues remained in G1, whereas fibroblasts mostly stayed in G2. This indicated that one of the mechanisms leading to EMT in CRSwNP may be that the proliferation ability of the epithelial cells was greatly weakened and that of the fibroblasts showed no significant changes, indicating that the increase in fibroblasts was not due to proliferation but possibly due to the transformation from epithelial cells. We also revealed that the proliferative capacity of the apical cells was reduced in the NP group than in the other two groups, which may be the reason for the obvious decrease in the proportion of apical cells. The decrease in the expression of ADGRB3 in the fibroblasts during CRSwNP formation may be due to causes other than their decreased proliferative capacity. The increase in POSTN+ fibroblasts during CRSwNP formation may have occurred because of EMT, which is formed by epithelial cell transformation. However, the specific mechanism needs to be explored in depth in future studies.

In addition to exploring cell subsets, the genes related to the formation and development of CRSwNP from the perspective of single cells were also investigated. Hao et al. discovered the adjacent middle turbinate mucosa and nasal polyps had a comparable immunohistochemistry pattern of mucosal inflammation, which pointed to a diffuse mucosal involvement, and this could account for the high incidence of recurrent nasal polyps attributing to the diseased middle turbinate mucosa^38^. Nasal polyps had a greater inflammatory infiltration than the nasal mucosa tissues adjacent to nasal polyps^39^. It suggested that the mucosa adjacent to nasal polyps also had inflammation, but the degree of inflammation was lower than that of nasal polyps, which might be a prepolypoid change. Therefore, our study focused on the continuous changes of normal, inflammatory mucosa, and polyp tissues and helped us better understand how to go from normal mucosa to polypoid changes with the intermediate status. These drove us to explore the related essential genes involved in the continuous changing process. We compared the three groups in the form of CM vs. UT and NP vs. UT. It could better reflect the dynamic change process. By comparing the differential genes between the NP vs. UT and UT vs. CM in epithelial cells and with the use of Venn diagram, 25 candidate genes were screened. Further screening revealed that WFDC2 was not only specifically expressed in epithelial cells but also showed a decreasing trend in the expression of CM, UT, and NP. WFDC2, also known as WAP four-disulfide core domain 2, is a member of the WFDC domain that functions as a protease inhibitor and has been confirmed to play an important role in colonic mucosal barrier homeostasis^40^. Nikoline et al. suggested that WFDC2 has been widely considered a potential tumour marker of epithelial ovarian cancer^41^. Tomazic et al. found that the expression level of WFDC2 was significantly downregulated in allergic rhinitis when compared to that in the normal population and that the downregulation of WFDC2 could increase the epithelial permeability of the mucous of the nasal cavity^42^. WFDC2 is crucial for the stabilization of mucosal epithelial cells; the gene expression level of WFDC2 is negatively correlated with lung function^43^. Epithelial–mesenchymal transition (EMT) was an essential process during cell development in which epithelial cells acquired mesenchymal fibroblast-like characteristics^8,44^. EMT was widely present in the nasal mucosa of CRSwNP and contributed to the pathogenesis of the disease^26^. However, the role of WFDC2 in pathogenesis has not yet been investigated. This study found that WFDC2 was downregulated in UT compared to that in CM and that it was further downregulated in NP groups. This study found that WFDC2 was downregulated in UT compared to that in CM and that it was further downregulated in NP groups. There was no difference in the positive rate of WFDC2 between eCRSwNP and neCRSwNP. This indicated that WFDC2 might inhibit the development of nasal polyps and there was no difference between eCRSwNP and neCRSwNP. It was mainly expressed in epithelial cells but not in fibroblast cells, indicating that WFDC2 might be a novel biomarker and be involved in the development of EMT in CRSwNP. The mechanism of WFDC2 in the development of CRSwNP deserved attention.

CCL26 has been identified as a marker of type 2 inflammation as well as a possible biomarker of CRSwNP^45^. The expression of CCL26 has a more obvious effect on the mechanism of eCRSwNP development. The mechanism of CCL26 in CRSwNP deserved further investigation. This provided us with ideas for future studies on the pathogenesis of CCL26 in eCRSwNP. EMT was an essential process in cell development in which epithelial cells acquired mesenchymal fibroblast-like characteristics and promoted the progression of inflammation^8,44^. Xia et al. found that many molecules were involved in the EMT process and promote the development of CRSwNP^26^. Our study indicated that CCL26 was found to be specifically expressed in fibroblasts, and its expression was increased in UT, and NP, respectively, compared to the CM group. It was mainly expressed in fibroblasts but not in epithelial cells, indicating that CCL26 might be an important biomarker and be involved in the development of EMT in CRSwNP. The expression of CCL26 was higher in NP(eCRSwNP) than in NP(neCRSwNP) groups. This suggested that CCL26 might promote the development of nasal polyps and played a more significant role in eCRSwNP. The specific mechanism of CCL26 in eosinophilic nasal polyps deserves to be explored further. The expression levels of WFDC2 and CCL26 were negatively correlated, indicating that they might have key roles in the occurrence and development of CRSwNP. This study has limitations and further study with a large sample size is needed to confirm our findings.

## Conclusions

In summary, this study mainly focused on the epithelial cells and fibroblasts in CRSwNP and analyzed their dynamics in three different status: nasal polyps, uncinate tissue adjacent to nasal polyps, and control nasal mucosa from patients with deviated nasal septa. The results have revealed the key cell subsets that may be involved in forming nasal polyps and nasal mucosa inflammation and identified WFDC2 and CCL26 as the key genes involved in developing CRSwNP. These two genes may serve as molecular targets in future CRSwNP treatments.

### Ethics approval and consent to participate

This study was approved by the Institutional Review Board of Beijing Chaoyang Hospital Ethics Committee (No. 20-department-52).

### Supplementary Information

Supplementary Information 1.

Supplementary Information 2.

## Data Availability

The original contributions presented in the study are included in the article/supplementary material. Further inquiries can be directed to the corresponding author.
