# Supplementary material for: Single-cell RNA sequencing reveals the epithelial cell, fibroblast, and key gene alterations in chronic rhinosinusitis with nasal polyps
Source: Sci Rep. 2024 Jan 27;14:2270. doi: 10.1038/s41598-024-52341-8 (PMC10821928; doi:10.1038/s41598-024-52341-8)
Supplement: Supplementary file 1 — Supplementary Information 1. [file 41598_2024_52341_MOESM1_ESM.docx]

**Sequencing library preparation and Single-cell RNA-seq data preprocessing**

We used Chromium Next GEM Single Cell 3ʹ Reagent Kits v3.1 on the Chromium Controller (10× Genomics) to prepare single-cell RNA-seq libraries. The individual cells of a microfluidic chip require reagents to stimulate reactions, and in this study, gel beads with cell barcode sequences wrapped in oil droplets were used to generate gel beads in the emulsion (GEMs). In the GEM oil droplets, the cells were lysed to release RNA, the RNA binds to a poly (dT) primer with the cell barcode Unique Molecular Identifiers (UMI) for complementary strand extension and adds 3 C bases to the end of the extended strand. The CCC end then completes the reverse transcription reaction by complementarily pairing with the rGrGrG of the Template switch oligo (TSO)and then uses the TSO as a template; subsequently, the GEMs are broken, recovered, and then enriched using PCR amplification of the cDNA for cDNA library construction. And the cDNA was recovered using magnetic beads (SPRI select Reagent（60ml）B). The full-length cDNA was then amplified by PCR and purified. The cDNA was quality controlled for concentration using Qubit 4.0 (Thermo Fisher Scientific) and integrity using Agilent 2100 (Aligent Technologies 2100 Bioanalyzer 2100 Expert, Version B.02.10.SI764). Library construction consisted of fragmentation, end repair, A-tailing, splice ligation, and index amplification. The library concentration was quality controlled using Qubit 4.0, and the library fragments were quality controlled using Qseq400 (SW Version: 3.4.0.0.6132, PID: 15420). Sequencing was performed on the Illumina NovaSeq 6000 with a sequencing depth of at least 50,000 reads per cell and 150 bp (PE150) paired-end reads (performed by Biomarker Technologies Corporation, Beijing, China).

10x Cell Ranger v7.0 was used to analyze the sequencing data. The Cell Ranger software called STAR (15) was used to statistically match Read2 to the reference genome. Based on the matching results from the STAR analysis and the information in the reference dataset (gtf/gff files), the read coverage information for each region on the genome was counted. The reads that matched known transcripts and were on the same strand were used for comparison with the transcripts. If the reads are compared to a known individual gene, the reads are referred to as unique compared to the transcriptome and only reads that are compared to the transcript can be counted as UMI. Cell Ranger removed the duplicate UMIs for each gene in each barcode and counted the number of unique UMIs to determine the gene expression in each cell.
