# Supplementary material for: Single-cell RNA sequencing reveals the epithelial cell, fibroblast, and key gene alterations in chronic rhinosinusitis with nasal polyps
Source: Sci Rep. 2024 Jan 27;14:2270. doi: 10.1038/s41598-024-52341-8 (PMC10821928; doi:10.1038/s41598-024-52341-8)
Supplement: Supplementary file 2 — Supplementary Information 2. [file 41598_2024_52341_MOESM2_ESM.docx]

**Supplementary Table 1** Clinical characteristics of control subjects and CRSwNP patients for 10x genomics scRNA-seq.

| Characteristics | CRSwNP (n=3) | Control (n=3) | *P* value |
| --- | --- | --- | --- |
| Age, median (yrs, IQR) | 41.0±8.6 | 42.0±3.6 | 0.887 |
| Sex (M/F) | 2/1 | 1/2 | 1.000 |
| Percentage of blood EOS (%, IQR) | 10.6±1.8 | 2.1±0.3 | 0.003 |
| Blood eosinophil count (10^9^ cells/L) | 0.90±0.10 | 0.12±0.03 | <0.001*** |
| Tissue eosinophil count/HPF | 102.7±35.7 | 7.0±1.6 | 0.019 |

Abbreviations: CRSwNP, chronic rhinosinusitis with nasal polyps; scRNA-seq, single-cell RNA sequencing; IQR, interquartile range; M, male; F, female; EOS, eosinophils; HPF, high-power field; *** *P* < 0.001

Details of clinical characteristics of control subjects and CRSwNP patients for 10x genomics scRNA-seq were summarized in Supplementary Table 1. According to the inclusion and exclusion criteria, six patients were enrolled, including the CRSwNP group (n=3) and the control group (n=3). The median age was 41.0±8.6 and 42.0±3.6, respectively. There were 2 males and 1 female in the CRSwNP group, while 1 male and 2 females in the control group. A significant difference was found in the percentage of blood EOS (%, IQR) (*P*=0.003), Blood eosinophil count (10^9^ cells/L) (*P*<0.001), and Tissue eosinophil count (*P*=0.019) between the two groups.

**Supplementary Table 2** Clinical characteristics of control subjects, eCRSwNP and neCRSwNP patients for IHC.

| Characteristics | eCRSwNP (n=11) | neCRSwNP (n=9) | Control (n=20) | *P* value |
| --- | --- | --- | --- | --- |
| Age, median (yrs, IQR) | 39.4±9.7 | 37.3±7.2 | 39.1±11.0 | 0.891 |
| Sex (M/F) | 5/6 | 5/4 | 11/9 | 0.738 |
| Percentage of blood EOS (%, IQR) | 11.9±2.6 | 1.9±0.7 | 2.0±0.9 | <0.001*** |
| Blood Eosinophil Count (10^9/L) | 0.98±0.25 | 0.12±0.07 | 0.13±0.07 | <0.001*** |
| Tissue Eosinophil Count/HPF | 127.5±52.0 | 5.8±2.6 | 6.0±2.4 | <0.001*** |

Abbreviations: CRSwNP, chronic rhinosinusitis with nasal polyps; eCRSwNP, eosinophilic chronic rhinosinusitis with nasal polyps; neCRSwNP, noneosinophilic chronic rhinosinusitis with nasal polyps; IHC, Immunohistochemistry; IQR, interquartile range; M, male; F, female; EOS, eosinophils; HPF, high-power field; *** *P* < 0.001

Details of clinical characteristics of control subjects, eCRSwNP, and neCRSwNP patients for IHC were summarized in Supplementary Table 2. According to the inclusion and exclusion criteria, forty patients were enrolled, including the eCRSwNP group (n=11), neCRSWNP group (n=9), and control group (n=20). The mean age was 39.4±9.7, 37.3±7.2, and 39.1±11.0, respectively. There were 5 males and 6 females in the eCRSwNP group, 5 males and 4 females in the neCRSwNP group, and 11 males and 9 females in the control group. Significant differences were found in the percentage of blood EOS (%, IQR), Blood eosinophil count (10^9^ cells/L), and Tissue eosinophil count among the three groups (*P*<0.001).
